# Chemical-genetic profile analysis of five inhibitory compounds in yeast

**DOI:** 10.1186/1472-6769-10-6

**Published:** 2010-08-06

**Authors:** Md Alamgir, Veronika Erukova, Matthew Jessulat, Ali Azizi, Ashkan Golshani

**Affiliations:** 1 Department of Biology, Carleton University, 1125 Colonel By Drive, Ottawa, K1 S 5B6, ON, Canada; 2 Ottawa Institute of System Biology, University of Ottawa, 451 Smyth Road, Ottawa, K1 H 8M5, ON, Canada; 3 Faculty of Medicine, University of Ottawa, 401 Smyth Road, Ottawa, K1 H 8L1, ON, Canada

## Abstract

**Background:**

Chemical-genetic profiling of inhibitory compounds can lead to identification of their modes of action. These profiles can help elucidate the complex interactions between small bioactive compounds and the cell machinery, and explain putative gene function(s).

**Results:**

Colony size reduction was used to investigate the chemical-genetic profile of cycloheximide, 3-amino-1,2,4-triazole, paromomycin, streptomycin and neomycin in the yeast *Saccharomyces cerevisiae*. These compounds target the process of protein biosynthesis. More than 70,000 strains were analyzed from the array of gene deletion mutant yeast strains. As expected, the overall profiles of the tested compounds were similar, with deletions for genes involved in protein biosynthesis being the major category followed by metabolism. This implies that novel genes involved in protein biosynthesis could be identified from these profiles. Further investigations were carried out to assess the activity of three profiled genes in the process of protein biosynthesis using relative fitness of double mutants and other genetic assays.

**Conclusion:**

Chemical-genetic profiles provide insight into the molecular mechanism(s) of the examined compounds by elucidating their potential primary and secondary cellular target sites. Our follow-up investigations into the activity of three profiled genes in the process of protein biosynthesis provided further evidence concerning the usefulness of chemical-genetic analyses for annotating gene functions. We termed these genes *TAE2*, *TAE3 *and *TAE4 *for translation associated elements 2-4.

## Background

Predicting gene function is a major goal of systems molecular biology in the post genome sequencing era. In this context, the yeast *Saccharomyces cerevisiae *has emerged as the eukaryotic model organism of choice for large-scale functional genomic investigations. Yeast cells have been subjected to a number of high throughput investigations such as gene expression analysis [1], protein-protein interaction mapping [2,3] and synthetic genetic interaction analysis [4]. Much knowledge relating to the functions of yeast genes has been collated but a significant number of genes are still not characterized in this model organism [5]. Consequently, further studies are required to examine the function(s) of uncharacterized genes, and to investigate novel function(s) for genes that are not fully characterized.

Increased sensitivity of gene deletion mutant strains to inhibitory compounds has been used extensively to study gene functions [6,7]. This approach is partly based on the theory that in general, the presence of redundant pathways compensates for genetic inactivation of a single pathway, with no phenotypic consequence [8]. However, the inactivity of a second functionally overlapping pathway, in this case using a chemical treatment, can cause a "double hit" effect and result in a phenotypic consequence that can be scored as a reduction in the rate of growth, or a sick/sensitive phenotype [4,9]. Similarly, such chemical-genetic profile analyses can be used to study cellular target sites of various bioactive compounds [9], pharmaceuticals [10] and herbal extracts [11] whose mechanisms of action are unknown.

In general, chemical sensitivity profiling of yeast gene knockouts can be studied using three complementary high throughput approaches. In the first case, deletion mutants can be grown individually in liquid cultures and their growth rates monitored spectrophotometrically using a microplate reader. The growth curve of micro-cultivated mutant strains in the presence and absence of a bioactive compound is used to determine strain sensitivity [12-14]. The second approach is based on synthetic lethality analysis on microarray (SLAM) [15]. A pool of tagged deletion strains are grown in the presence and absence of the target compounds. Owing to the presence of a specific barcode in each mutant strain, the relative growth of each strain can be determined using microarray methodology, and sensitivity is measured on the basis of the relative growth of a specific mutant strain in the presence of other strains. The third approach concerns colonies of yeast gene deletion mutant strains being arrayed on solid media in the presence and absence of the target compounds [9,16]. The growth rates of individual colonies are estimated by their relative colony size relative to a control. Each of these techniques has inherent advantages and disadvantages. The results obtained from these methodologies are considered to be complementary [17].

Recently, we showed that high throughput chemical sensitivity profile analysis of yeast gene knockout strains to paromomycin can be used to study novel gene functions, and reported that a previously uncharacterized open reading frame, *TAE1*, has a novel role in protein biosynthesis or translation [18]. In the present study, colony size reduction was used to screen and analyze the yeast gene knockout collection for their sensitivity to five bioactive compounds that target the process of protein biosynthesis. We followed up by studying the activities of three profiled genes for their involvements in protein biosynthesis and termed them *TAE2*, *TAE3 *and *TAE4 *for translation associated elements 2-4.

## Results

### Drug sensitivity screens

The entire collection of the haploid yeast gene deletion array (yGDA) (~4700) was screened for increased sensitivity to the bioactive compounds cycloheximide, 3-amino-1,2,4-triazole (3-AT), paromomycin, streptomycin and neomycin. These drugs have reported involvement in protein biosynthesis or translation. Cycloheximide is a glutarimide antibiotic that binds to the 60 S ribosomal subunit and inhibits translation elongation [19]. 3-AT is a competitive inhibitor of imidazole glycerol phosphate dehydratase, an enzyme involved in the biosynthesis of the amino acid histidine [20], and causes amino acid starvation [21]. Paromomycin, neomycin and streptomycin are known to bind the small ribosomal subunit of eukaryotic cells, inhibit ribosomal translocation and compromise translation fidelity [22]. We hypothesized that sensitivity to these drugs could be used as a method of identifying new genes associated with the process of protein biosynthesis. Sub-inhibitory concentrations of the drugs were used in this study. Under these growth conditions only strains with increased sensitivity would demonstrate growth reduction and the growth of the remaining strains would be largely unaffected. Each experiment was repeated three times and the total number of analyses exceeded 70,000. We have previously reported analysis concerning sensitivity to paromomycin [18]. Here, we present the collective analysis of the total data from the entire collection of sensitive strains.

Colony size (CS) measurement was used to determine sensitivity. The relative colony growths (normalized to the average growth on the plate) on treated plates were compared with those grown under control conditions (untreated) as described previously [23]. CS measurement is an established method used to identify drug sensitive strains [9,16,24] and is reported to identify approximately 63% of sensitive strains detected by standard large-scale spot test (ST) analysis [23]. Therefore, a number of sensitive strains that could be detected using ST analysis may not have been detected using CS, indicating that our approach using CS to identify sensitivity is not exhaustive. Furthermore, it is reported that 59% of the sensitive strains detected by CS are not detected by ST and hence may represent novel/false positives.

To reduce false positive results, gene deletion strains with sensitivity to different unrelated bioactive compounds that we continuously observed in our previous independent screens, as well as those reported by others [25], were eliminated from the list of sensitive strains. These genes typically represent multiple-drug resistant genes that are not linked to the cellular target sites of the drugs of interest. Inclusion of these genes would complicate the analysis of the molecular activity of the target compounds. The final list of the genes that when deleted increased drug sensitivity to the tested bioactive compounds is presented in Additional file 1. There are 383, 320, 205, 99 and 89 genes that, when deleted, confer increased sensitivity to cycloheximide, 3-AT, streptomycin, neomycin and paromomycin, respectively. These are non-essential genes that are normally not required for the growth of yeast cells under typical laboratory conditions, suggesting that the slow growth phenotype of the corresponding deletion strains is a direct result of the inhibitory effects of the target drugs. Cycloheximide, streptomycin, neomycin and paromomycin bind to ribosomes and cause defects in protein synthesis [26,27]. Therefore, as expected, among gene deletion strains with increased sensitivity to these drugs, we identified numerous previously characterized protein synthesis related genes including ribosomal protein L27A gene *YHR010W *(*RPL27A*), translation initiation factor *YJL138C *(*TIF2*), tRNA methyltransferase *YDL201W *(*TRM8*), mitochondrial translation initiation factor *YOL023W *(*IFM1*) and eIF4E-associated gene *EAP1 *(*YKL204W*).

**Figure 1:**
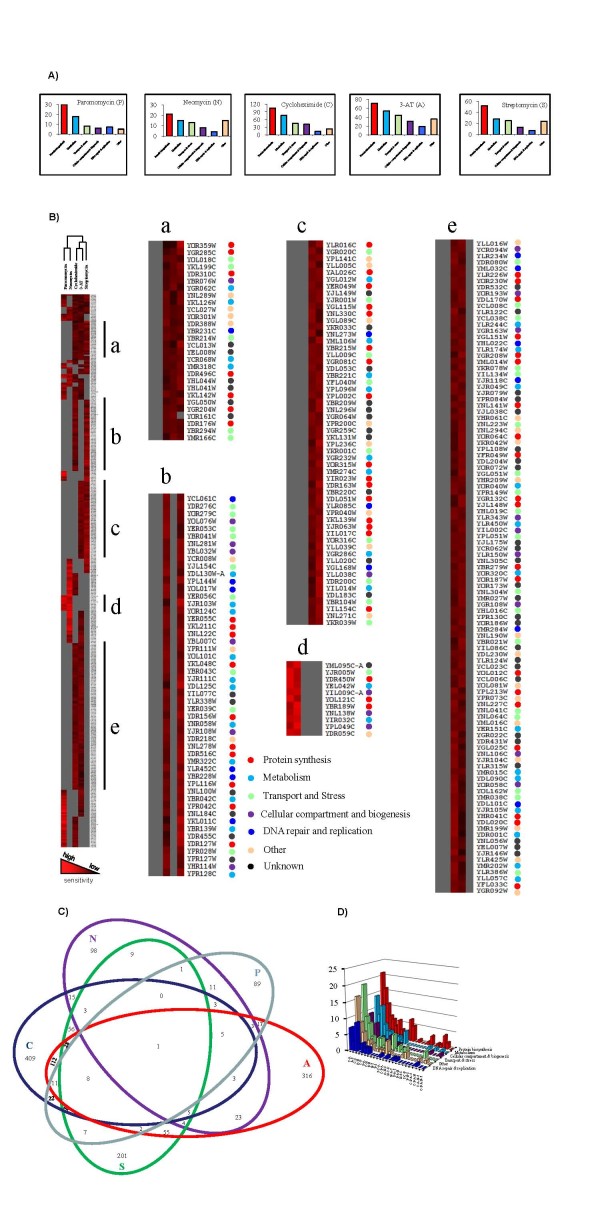
**Clustering of drug sensitive gene deletion mutants**. The haploid non-essential yeast gene deletion array was subjected to sub-inhibitory concentrations of five inhibitory compounds. Colony size reduction was used to detect sensitivity. (A) Drug sensitive yeast gene deletion mutants were clustered according to the cellular processes in which their deleted genes participated. The overall distributions of gene functions were comparable for different treatments with protein biosynthesis as a major group for all treatments. (B) Chemical profiles were clustered according to drug sensitivities to two or more drugs. Hierarchical clustering of mutants is illustrated using complete linkage. Absolute correlation coefficient (centered) is used for comparability and displayed in Java TreeView. Several regions of interest (a-e) are enlarged. The cellular processes of the deleted genes are color-coded. On the basis of sensitivity profiles, paromomycin is grouped with neomycin. Cycloheximide is grouped with 3-AT, which then merges with streptomycin. Sensitivity indexes of the gene deletion mutants are shown as high to low (light to dark red). (C) Sensitivity overlaps for gene deletion mutants to different drug treatments. The number of gene deletion mutants with a particular sensitivity, for example paromomycin (P) alone (89), paromomycin and 3-AT (17) and paromomycin, 3-AT and neomycin (3), are indicated. (D) The overlapping drug sensitive yeast gene deletion mutants are clustered according to the cellular processes in which their deleted genes participate. No significant enrichment for protein biosynthesis genes among overlapping sensitive strains was observed. The number of sensitive strains is presented on the z-axis. C: cycloheximide; P: paromomycin; A: 3-AT; N: neomycin; and S: streptomycin. The sensitivity overlaps between P and N, C and 3-AT, C and S, and 3-AT and S were significant with *P*-values ≤ 5 × 10^-14^. Other overlaps are significant with *P-*values of ≤ 0.029.

Excluding genes with unknown functions, the clustering of the identified genes based on the cellular processes in which they participate is presented in Figure 1a (also see Additional file 1). The dominant clusters are protein biosynthesis related genes. For example, approximately 33% of strains sensitive to cycloheximide are linked to protein biosynthesis; approximately 24%, 15%, 13%, 5% and 10% are associated with metabolism, cellular compartments and biogenesis, transport and stress, DNA repair and replication, and others, respectively. This was expected as cycloheximide, streptomycin, neomycin and paromomycin are known to interact directly with ribosomal subunits. 3-AT also affects protein biosynthesis by altering the available pool of amino acids [20,21].

Smaller clusters could represent additional target sites (side effects) of the drugs. For example, neomycin is known to inhibit the phospholipase C pathway and thus interfere with signal transduction in eukaryotic cells [28]. This could explain the observation that deletion of *YIL050W *(*PCL7*), which codes for a member of a metabolism-associated Pho85c kinase complex, confers cell sensitivity to neomycin. The smaller clusters could also represent novel secondary functions for certain genes, some of which may link translation to other cellular processes. For example, deletion of *YER095W *(*RAD51*) or *YOL090W *(*MSH2*) increased sensitivity to cycloheximide. *YER095W *and *YOL090W *are involved in repair of DNA strand breaks. Interestingly, *YER095W *is reported to have a genetic interaction (positive genetic) with the translation termination factor eRF3 gene *YDR172W *(*SUP35*) and the translation elongation factor *YLR249W *(*YEF3*) gene [29], and its product is reported to interact physically with glutamyl tRNA synthetase protein, *YGL245Wp *(*Gus1p*) [30]. Similarly, the gene product of *YOL090W *is reported to interact physically with the translation initiation factor eIF4A, *YJL138C *(*TIF2*) [31]. This is in agreement with the recently reported link between DNA damage response and translation [32]. Alternatively, the smaller clusters could represent false positive results. However, the most likely scenario is that each of the aforementioned cases represents a different integrated part of the data. For example, secondary target sites of a drug can be investigated with the prior knowledge that the smaller clusters could contain genes with novel secondary functions as well as a number of false positives. An interesting observation is that the overall distribution of genes within each functional cluster was similar for each of the five drugs investigated herein (Figure 1A). This could represent cross-talk between protein synthesis and the other four cellular processes. Based on our previous observations of chemical-genomic profiles of other inhibitory compounds with diverse modes of action such as calcoflour white, methyl methane sulfate, and sodium dodecyl sulfate, the profiles presented in Figure 1A are distinct (unpublished data).

As utilized previously [33], a hierarchical clustering approach to drug sensitivity was used to analyze the chemical profiles (Figure 1B). It is expected that compounds with similar modes of activity have similar profiles with considerable overlaps, and hence cluster together. As expected, the profiles for paromomycin and neomycin had considerable overlaps and hence these compounds were clustered together by hierarchical clustering using complete linkage. These aminoglycosides bind small ribosomal subunits and compromise translation fidelity and translocation. Cycloheximide and 3-AT also had considerable overlaps and were clustered together as expected; these drugs can affect the elongation phase of translation. Cycloheximide does so by binding the 60 S ribosomal subunit [19] whereas 3-AT causes starvation of amino acids needed for successful elongation. Interestingly, streptomycin, an aminoglycoside, had more overlap and was more closely associated with cycloheximide and 3-AT. Unlike other aminoglycosides, streptomycin does not bind the ribosomal A-site [34], implying that streptomycin binding to the ribosome could result in an alternative ribosomal conformation that resembles the action of cycloheximide and 3-AT. The effect of streptomycin on prokaryotic translation elongation, which is different from other aminoglycosides, is well documented [35].

The overlap of strain sensitivities to different drugs is represented in Figure 1C. A total of 1519 gene deletion mutants were identified with increased sensitivity to a minimum of one drug (Figure 1C); 408 were sensitive to two or more drugs. A mutant for the vacuole gene *YDR495CΔ *(*vps3Δ*) was sensitive to the five treatments. This mutant has been observed in other screens, suggesting non-specific involvement in multiple drug resistance. When analyzing the overlapping drug sensitive strains, the ratio of protein synthesis related genes did not increase significantly when sensitivities to two or more drugs were analyzed (Figure 1C and 1D and Additional file 1). Enrichment in the category of transport and stress related genes, into which multiple drug resistant genes generally fall, was observed for some multiple drug sensitive groups. This highlights that selection based on several drugs could partially target multiple drug resistant genes.

**Figure 2:**
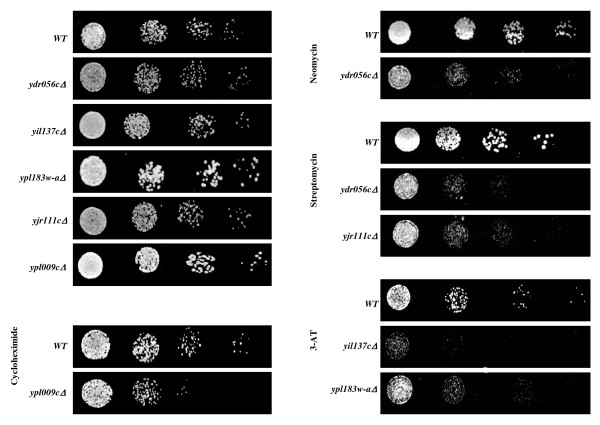
**Strain sensitivity to different translation-inhibitory drugs**. Wild type (WT) or gene deletion mutant strains (*yploo9cΔ*, *yil137cΔ*, *ypl183w*-*a*Δ, *ydr056cC*Δ and *yjr111cΔ*) were serially diluted to 10^-3 ^to 10^-6 ^and spotted on solid medium with sub-inhibitory concentrations of cycloheximide, paromomycin, 3-AT, streptomycin and neomycin as indicated, or without drugs (control). The plates were incubated at 30°C for 1-2 days. Deletion of *ypl009c *confers increased sensitivity to cycloheximide; *yil137c *and *ypl183w-a *to 3-AT, *ydr056c *to streptomycin and neomycin, and *yjr111c *to streptomycin.

To investigate the accuracy of our large-scale approach to detect drug sensitive mutants, five deletion strains were selected and subjected to spot test analysis (Figure 2). This analysis confirmed that deletion of *YPL009C *confers increased sensitivity to cycloheximide, deletion of *YDR056C *increases sensitivity to streptomycin and neomycin, deletion of *YJR111C *increases sensitivity to streptomycin, and deletions of *YIL137C *and *YPL183W*-A increase sensitivity to 3-AT. These results are in agreement with the large-scale analysis and confirm that this approach can identify strains that are sensitive to the drugs used in this study.

### Synthetic genetic array (SGA) analysis for *TAE2*, *TAE3 *and *TAE4*

The majority of mutants with increased sensitivity to the target drugs had deletions of genes with known functions in protein biosynthesis. Therefore, the activities of three mutants for genes that are not well characterized, *YPL009C*, *YIL137C *and *YPL183W*-A, were examined by studying genetic interactions with previously reported protein biosynthesis related genes. These genes have not been characterized but available literature and our unpublished data suggest possible associations with certain disease related-genes and phenotypes (see Discussion).

**Figure 3:**
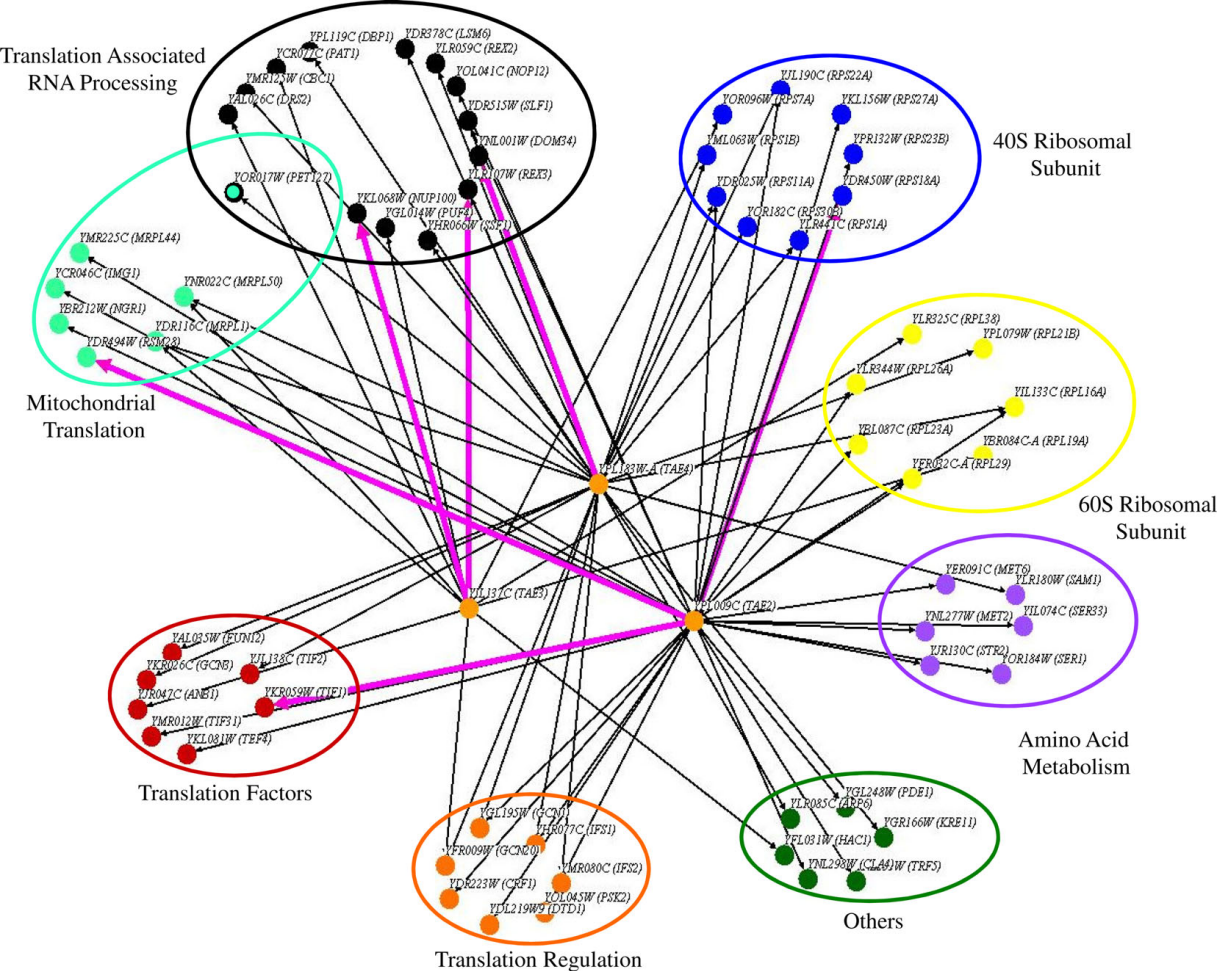
**Synthetic genetic interaction analysis for *TAE2*, *TAE3 *and *TAE4 *with translation related genes**. There are 72 interactions that represent synthetic genetic interactions for three query genes *TAE2*, *TAE3 *and *TAE4*, with 59 different translation genes. Genes are represented as nodes (circles) and interactions are represented as edges (lines). The interacting genes are further divided into eight functional categories. There are a number of shared interactions that highlight the interconnectivity of the network. The nodes are coloured according to functional groups. Black edges represent synthetic sick (aggravating) interactions, and the six pink thick edges represent synthetically rescue (alleviating) interactions.

It is generally accepted that many genes/pathways in eukaryotic cells are functionally redundant and that compensation for loss of activity is prevalent [8]. However, deletion of a second functionally related gene/pathway could result in sickness or lethality, indicating an aggravating interaction. Consequently, the sickness of double mutants can be used to investigate genetic interaction and functional relationships between genes (synthetic genetic interaction analysis) [4]. The synthetic genetic interactions of *YPL009C*, *YIL137C *and *YPL183W*-A with other protein biosynthesis genes were investigated by systematically examining double gene deletions for alterations in colony size [4]. If our targeted genes are involved in protein biosynthesis, it would be expected on the basis of their molecular function that they would interact genetically with other translation genes with related functions. As presented in Figure 3 and Additional file 2, *YPL009C*, *YIL137C *and *YPL183W*-A interacted genetically with a number of translation genes as evidenced by the sick phenotype of the double mutants. These results suggest a functional association for our target genes with the process of protein biosynthesis. Therefore, the studied genes were named *TAE2 *(*YPL009C*), *TAE3 *(*YIL137C*) and *TAE4 *(*YPL183W-A*), or translation associated elements 2-4, respectively. The largest group of genes that interacted with *TAE3 *and *TAE4 *were those involved in translation associated RNA processing, with three and seven interactions, respectively. This group included genes such RNA exonuclease *YLR059C *(*REX2*), which is involved in rRNA maturation and processing, rRNA binding protein YHR066Wp (Ssf1p), which is a constituent of the 66 S pre-ribosomal subunit, and nuclear pore complex protein YKL068Wp (Nup100p), which is involved in mRNA and rRNA export and ribosomal protein import to the nucleus. *TAE4 *interacted with five genes related to different small ribosomal subunit proteins including *YLR441C*, which codes for S1A, and *YJL190C*, which codes for S22A. *TAE2 *had a general pattern of interaction and interacted with genes with differing functions. The largest groups of genes (three) that interacted with *TAE2 *had five members each, with functions in amino acid biosynthesis, small ribosomal subunit proteins and regulation of translation.

In addition, some of the identified genetic partners were shared between the query genes (Figure 3). For example, *YDR025W*, which codes for the small ribosomal subunit protein S11A, interacted genetically with *TAE2 *and *TAE4*, and *YFR009W *(*GCN20*), which is involved in positive activation of GCN2 kinase, interacted with *TAE3 *and *TAE4*. Furthermore, a synthetic genetic interaction between *TAE2 *and *TAE4 *was observed.

In contrast to the aggravating interactions in which sickness of double mutants was investigated, interactions concerning double mutants with higher fitness than expected were examined. Such alleviating interactions, also known as synthetic rescue, are thought to exist between genes in the same pathway [36]. Six such interactions were identified in this study (Figure 3). In agreement with the synthetic sickness interactions, which showed that the largest functional interaction partners for *TAE3 *and *TAE4 *were involved in translation associated RNA processing, it was observed that *TAE3 *interacted with the RNA processing gene *YLR107W *(*REX3*) and with *YKL068W *(*NUP100*), a gene involved in RNA transport from the nucleus and associated with rRNA and tRNA export, and that *TAE4 *interacted with another RNA processing gene, *YNL001W *(*DOM34*). *TAE2 *had alleviating interactions with three genes with different functions, namely *YKR059W *(*TIF1*), which has a role in translation initiation, *YDR494W *(*RSM28*), which is involved in mitochondrial translation, and *YDR450W *(*RPS18A*), associated with the structure of small ribosomal subunits. The diversity of the interactions in which *TAE2 *is involved mirrors the results of the synthetic sick interactions, leading to the conclusion that it did not interact with one major functional group.

A recent genome-wide synthetic genetic interaction study used *TAE3 *and *TAE4 *as query genes and demonstrated that they formed synthetic sick and lethal interactions predominantly with genes involved in protein biosynthesis (*P*-values of 10^-7 ^and 10^-16 ^for *TAE3 *and *TAE4*, respectively) [37], confirming the results presented herein. Similarly, the synthetic sick and lethal interactions reported for *TAE2 *predominantly (*P*-value = 0.003) concerned protein biosynthesis genes.

### Functional correlations for *TAE2 *and *TAE4 *with other protein synthesis related genes

**Figure 4:**
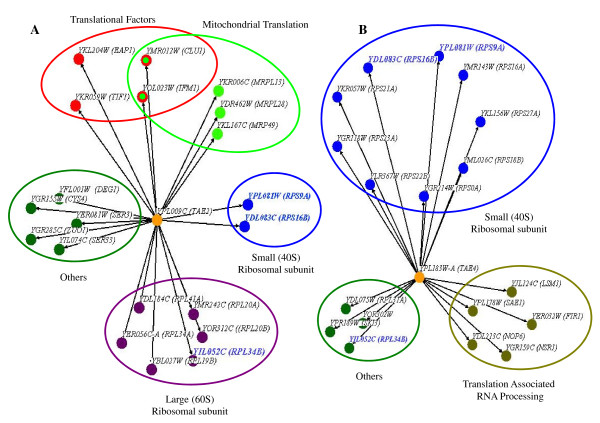
**Overexpression of *TAE2 *and *TAE4 *suppresses the sensitivity of numerous translation genes to drug treatments**. Overexpression of *TAE2 *and *TAE4 *suppresses the phenotype of a number of translation gene deletion strains against neomycin and/or streptomycin treatments. Genes are represented as nodes (circles) and interactions are represented as edges (lines). The interacting genes are divided into functional categories and colored accordingly. (A) *TAE2 *over-expression rescued 20 gene deletions with a variety of functions. (B) *TAE4 *over-expression rescued 18 gene deletions, the majority of which are 40 S subunit proteins (nine genes) or function as translation-associated RNA processing proteins (five genes). Blue letters represent genes that are rescued by the overexpression of both *TAE2 *and *TAE4*.

Overexpression of a gene often compensates for a phenotypic consequence caused by the absence of a functionally related gene [38,39]. Therefore, one approach to studying protein function would be to investigate whether its overexpression can compensate for the absence of proteins with known functions. This approach was used to investigate further the biological activity of the gene products for *TAE2 *and *TAE4 *by investigating whether their overexpression could reverse the phenotypic consequences caused by the absence of other translation genes (phenotypic suppression analysis). For an unknown reason our multiple attempts to isolate an overexpression plasmid for *TAE3 *from the yeast gene overexpression library were unsuccessful. Consequently, *TAE3 *was omitted from this part of the investigation. Reduced growth was used as the target phenotypic consequence for gene deletion strains cultured in the presence of neomycin and streptomycin. As indicated in Figure 4 (and Additional file 3), we observed that the growth defects in the presence of neomycin and/or streptomycin for a number of deletion strains for translation genes were compensated by the overexpression of *TAE2 *(Figure 4A) or *TAE4 *(Figure 4B). In agreement with the synthetic genetic interactions described previously, the two main functional categories that *TAE4 *overexpression rescued included genes involved in translation related RNA processing and 40 S ribosomal structure maintenance. For example, *TAE4 *overexpression rescued the sensitivity to drugs of deletion strains for the pre rRNA processing gene *YGR159C *(*NSR1*) and the 40 S ribosomal subunit protein S28 gene *YGR118W *(*RPS23A*). These observations can be explained by a role for *TAE4 *in 40 S biogenesis, which is in agreement with the synthetic sick and synthetic rescue interactions observed for *TAE4*.

As was the case with the synthetic genetic interactions for *TAE2*, the phenotypic suppression analysis suggested a general role for *TAE2 *in translation. Overexpression of *TAE2 *compensated for the deletion of a number of genes with diverse roles in translation such as *YMR242C *(*RPL20A*), which codes for a 60 S ribosomal subunit protein, *YDR462W *(*MRPL28*), which codes for a mitochondrial ribosome protein, and *YKR059W *(*TIF1*), which codes for the translation initiation factor eIF4A.

Three of the rescued gene deletion strains, *YDL083CΔ *(*rps16BΔ*), *YPL081W*Δ (*rps9AΔ*) and *YIL052C*Δ (*rpl34BΔ*), were shared between *TAE2 *and *TAE4*. This is in accordance with the synthetic genetic interaction observed between these two genes (Figure 3). Such interactions highlight the interconnectivity of a genetic interaction map for translation genes.

### Deletions of *TAE2*, *TAE3 *and *TAE4 *affect the process of protein synthesis

The genetic interaction analyses provide a direct link between *TAE2*, *TAE3 *and *TAE4*, and the process of protein biosynthesis. To investigate this link further we examined the effect of deletion of the target genes on translation efficiency, stop codon readthrough and ribosome biogenesis. If any differences were detected we would expect them to be subtle owing to the importance of protein biosynthesis for cell survival and the fact that the deletion of the target genes does not change the growth rate of the mutants under standard laboratory conditions.

**Figure 5:**
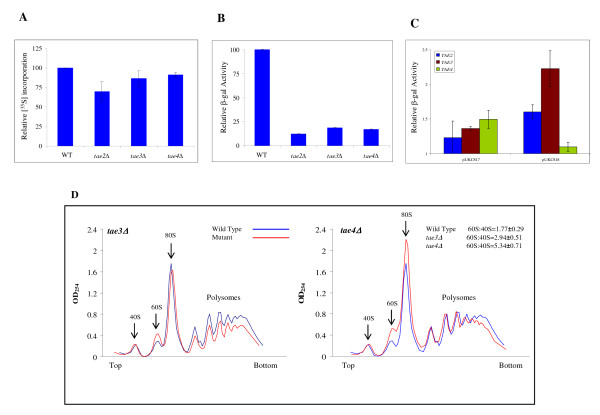
**Characterization of *TAE2, TAE3 *and *TAE4 *deletions**. (A) Total protein synthesis was measured using [^35^S] methionine incorporation in wild type, *tae2Δ*, *tae3Δ *and *tae4Δ *strains. The average count for [^35^S] methionine incorporation for wild type was 11,356,073 (± 1,400,000) counts, which is set to 100%. On average, in the absence of Tae2p, Tae3p and Tae4p, [^35^S] methionine incorporation was reduced by approximately 30, 14 and 10%, respectively. (B) The efficiency of protein synthesis was measured using an inducible β-galactosidase reporter construct (p416). The average β-galactosidase activity for wild type was 7.5 (± 0.6) units, which is set to 100%. The β-galactosidase activity was measured after 4 h induction. Deletion of *TAE2*, *TAE3 *and *TAE4 *limited the expression of β-galactosidase to 13, 21 and 17% of that in wild type, respectively. (C) Deletion of *TAE2, TAE3 and TAE4 *resulted in increased levels of β-galactosidase from *lacZ *reporters with different premature stop codons (pUKC817 and pUKC818). The activity of β-galactosidase was determined by normalizing the activity of the mutant (pUKC817 and pUKC818) to the control (pUKC815). pUKC815 is the background construct without a premature stop codon and is used as a control. Bars represent standard deviations for the means. **(**D**) **Ribosome profile analysis of yeast deletion strains *tae3Δ *and *tae4Δ *compared to wild type. Deletion of *TAE3 *decreased the levels of polysomes and increased free 60 S subunits. Deletion of *TAE4 *caused an increase in free 60 S subunits and a slight decrease in larger polysomes. Each experiment was repeated a minimum of three times. Ratios of free 60S:40 S were calculated from the areas under the curves.

We first investigated the involvement of *TAE2*, *TAE3 *and *TAE4 *in translation efficiency. Deletion mutants *tae2*Δ, *tae3*Δ and *tae4*Δ were subjected to [^35^S] methionine incorporation analysis. *tae2*Δ, *tae3*Δ and *tae4*Δ mutant strains demonstrated approximately 30%, 14% and 10% reduced levels of [^35^S] methionine incorporation, respectively (Figure 5A). To complement these findings, we investigated the rate of protein synthesis using an inducible β-galactosidase reporter construct (p416) under the control of a GAL1 promoter [40], which better highlights differences in translation efficiencies [18]. After four hours of induction, levels of β-galactosidase activity were six fold lower for *tae2*Δ and *tae3*Δ mutants, and five fold lower for *tae4*Δ (Figure 5B) while their mRNA contents remained relatively unchanged (data not shown).

A plasmid-based β-galactosidase system with different premature termination codons was used to study stop codon readthrough. In this approach alterations in translation fidelity lead to an increase in termination codon readthrough and thus elevate the production of full length functional β-galactosidase. To this end, target deletion strains were transformed with three different plasmids, pUKC815, pUKC817and pUKC818 [41], and the expression of β-galactosidase in each mutant was quantified. pUKC815 contains no in-frame premature termination codon and was used as a control. pUKC817 and pUKC818 contain in-frame termination codons UAA and UAG, respectively. Apparent from the increased relative productions of β-galactosidase shown in Figure 5C, deletion of *TAE2*, *TAE3 *and *TAE4 *resulted in higher levels of termination codon readthrough. Comparable levels of β-galactosidase mRNA were evident in each of the tested strains (data not shown) demonstrating that the observed increase s in β-galactosidase activity were not due to altered levels of mRNA.

A surprising observation was that deletion of *TAE4 *resulted in a higher readthrough for the UAA (pUKC817) stop codon but not UAG (pUKC818). Generally, it is expected that alterations in translation fidelity result in more readthrough for a less stringent stop codon, in this case UAG. This was observed for *tae2*Δ and *tae3*Δ but not *tae4*Δ. A possible explanation is that deletion of *TAE4 *causes an alteration that is stop codon specific. For example, it could reduce the affinity of ribosomes for a specific translation release factor (RF) but not others.

Next, the ribosome profiles for *tae2*Δ, *tae3*Δ and *tae4*Δ gene deletion strains were investigated. The profiles had three peaks associated with free 40 S and 60 S subunits and 80 S monosomes, followed by a series of peaks representing polysomes (Figure 5D). The ribosome profile for *tae2*Δ was comparable to the wild type strain (data not shown). However, for *tae3*Δ, a reduction in polysomes was observed, as was an increase in free 60 S subunit (Figure 5D). The free 60S:40 S subunit ratio for this mutant was 2.94 ± 0.51 in comparison to 1.77 ± 0.29 for the wild type. Similarly, the profile for *tae4*Δ demonstrated a significant increase in free 60 S subunits, a slight increase in 80 S monosomes and a slight reduction in larger polysomes (Figure 5D). The free 60S:40 S subunit ratio for *tae4*Δ was 5.34 ± 0.71. Reduction of polysomes could explain the observed reductions in the efficiency of protein synthesis for *tae3*Δ and *tae4*Δ. Alterations in the pool of free ribosomal subunits could relate to deficits in subunit biogenesis, suggesting that *TAE3 *and *TAE4 *could be involved in the process of ribosome biogenesis. The 40 S and 60 S subunits are in equilibrium with 80 S monosomes, therefore an increase in 60 S free subunits could relate to a defect in 40 S biogenesis [42] as observed for *tae3*Δ and *tae4*Δ mutants. A more precise calculation for measuring free 60S:40 S involves measuring 40 S and 60 S subunits separated on a sucrose gradient with low concentrations of Mg^2+^, but this was not carried out in the present study.

## Discussion

Gene deletions that cause increased sensitivity to a bioactive compound can be used to identify pathways that buffer the cell against the activity of that compound [43]. Therefore, chemical-genetic profiles of inhibitory compounds can lead to identification of their overall mode of action and any side effects associated with toxicity of the drug. Furthermore, these profiles can help identify novel genes involved in specific cellular pathways targeted by compounds [9]. In this study the sensitivity of a yeast gene deletion array to five different bioactive compounds was investigated using colony size reduction as the endpoint. The overall profiles of these compounds were comparable, with the deletion of genes involved in protein biosynthesis being the dominant cluster. Further investigations concerned three sensitive deletion strains for genes that are not well characterized, that here we term *TAE2*, *TAE3 *and *TAE4*. Genetic analyses provided evidence for involvement of these genes in protein biosynthesis.

There is limited information relating to the molecular activity of Tae4p. In a large-scale investigation it was reported that deletion of *TAE4 *rescued the temperature sensitivity of *cdc13-1*, so it was thought to be a restrictor of telomere capping; Cdc13p is an essential protein involved in checkpoint and telomere capping. The C-terminal domain of Tae4p, amino acids 56-93, has sequence homology to the prokaryotic ribosomal protein L36. In the current study it was observed that *TAE4 *formed synthetic sick interactions with two predominant categories of genes with functions in RNA processing associated with protein biosynthesis and genes that code for 40 S ribosomal subunit associated proteins. Furthermore, it formed phenotypic suppression interactions with the same two categories of genes, linking the activity of *TAE4 *to RNA processing and the 40 S subunit. TAE4 involvement in 40 S biogenesis was confirmed by ribosome profile analysis and explains the observed effects of the deletion of *TAE4 *on stop codon readthrough and the efficiency of translation. Furthermore, *TAE4 *is co-regulated with several rRNA processing proteins such as the LSM protein YJR022Wp (Lsm8p) implicated in pre-rRNA and pre-tRNA processing [44] and a preribosome processing protein YLR409Cp (Utp21p) involved in 18 S rRNA processing [45]. These observations, together with the results presented here, provide strong evidence that the activity of *TAE4 *is related to RNA processing and ribosome biogenesis. The C-terminal domain of Tae4p contains two RNA binding domains. Therefore, Tae4p can affect rRNA processing by directly binding to rRNA or by recruiting other factors to rRNA, which in turn can affect ribosome biogenesis.

*TAE2 *(*YPL009C*) has no previously reported cellular function but its protein product shares a domain similar to the human colon cancer antigen 1 (*SDCCAG1*) and has some sequence homology with a putative RNA binding protein in *Drosophila melanogaster*. The synthetic sick and synthetic rescue genetic interaction analyses presented herein indicated a diverse interaction pattern for *TAE2 *with various translation genes. *TAE2 *overexpression rescued the phenotype of deletion strains for genes with different functions in various steps of translation, suggesting a general involvement of *TAE2 *in protein biosynthesis that is not targeted to a specific pathway. Deletion of *TAE2 *caused an increase in stop codon readthrough and a decrease in translation efficiency. One possible explanation for these results is that *TAE2 *could transiently aid in mediating the overall activity of ribosomes and hence translation efficiency and fidelity. Translation *in vivo *is more efficient than in reconstituted *in vitro *experiments, indicating the presence of uncharacterized translation elements *in vivo *[46]. The activity of *TAE2 *is supported by the observation that in large-scale affinity purification experiments Tae2p co-purified with several ribosomal subunit proteins [30]. However, low concentrations of salt destabilize this interaction [47] suggesting that Tae2p transiently interacts with ribosomes. In addition, Tae2p was recently computationally predicted to interact directly with the ribosomal subunit protein YDR418Wp (Rpl2Bp) [48].

*TAE3 *predominantly formed synthetic sick interactions with RNA processing genes involved in translation and formed synthetic rescue interactions with genes with similar RNA processing functions. Alterations in the ribosomal profile of the *tae3*Δ strain suggest a deficiency in 40 S subunit biogenesis, and a role for *TAE3 *in RNA processing associated with 40 S biogenesis could explain these observations. In addition, this would explain the observation that deletion of *TAE3 *caused an increased stop codon readthrough and a reduction in the efficiency of protein synthesis. In agreement with this role for *TAE3*, the expression of *TAE3 *is reported to be strongly co-regulated with essential translation genes including *YAL003W *(*FUN53*), which codes for a subunit of RNase MRP involved in pre-rRNA cleavage, and *YBL004W *(*UTP20*), which is involved in 18 S rRNA processing [49]. Tae3p does not appear to contain an RNA binding domain. Therefore, it is possible that Tae3p may interact with an intermediate RNA binding protein(s) in order to exert its activity. In agreement with this, Tae3p was reported to co-purify with YOR272Wp (Ytm1p), a constituent of the 66 S pre-ribosomal particle [50]. In addition, we previously observed that deletion of *TAE3 *reduced the efficiency of double stranded DNA break repair, which could be an independent activity for *TAE3 *(unpublished data).

The effect of *TAE3 *deletion on stop codon readthrough is in agreement with a previous study [47]. However, the same investigation detected no apparent alteration in translation efficiency. A possible explanation for this difference is that the latter study utilized diploid homozygous gene deletion cells whereas the current study used haploid cells and the efficiency of induced translation was not measured in the former. Differences between the experimental observations in the haploid and diploid systems have been reported [51].

## Conclusion

The overall chemical-genetic profiles of the investigated compounds were comparable, with genes associated with protein biosynthesis being the dominant cluster, followed in a broad descending order by those involved in metabolism, cellular compartments and biogenesis, transport and stress, DNA repair and replication, and others. This observation may further underline previously speculated links between protein biosynthesis and other fundamental cellular processes. The smaller clusters could also represent alternative modes of activities for these compounds. Genetic investigations of three profiled genes further highlighted the effectiveness of chemical-genetic profiling for the investigation of gene functions for translation genes.

## Methods

### Growth media

Standard rich (YPD) and synthetic complete (SC) media were used for the experiments [52]. Yeast cells were grown at 30°C for 1-2 days. The YPD medium containing Geneticin (G418; 200 μg/ml) was used for the maintenance of deletion strains carrying the G418^r ^marker. To investigate the effects of drugs on the growth of yeast deletion mutants, paromomycin (10 mg/ml), streptomycin (40 mg/ml), neomycin (5.5 mg/ml) and 3-AT (22 mg/ml) were added to SC medium and cycloheximide (45 ng/ml) was added to YPD medium. G418 sulfate, cycloheximide and 3-AT were purchased from Sigma and paromomycin sulfate from Fluka. Neomycin sulfate and streptomycin were obtained from Bioshop, Canada.

### Drug sensitivity analysis

MIC for each compound was measured as the lowest drug concentration that resulted in inhibition of visible growth of yeast strains on sterile 96-well microtitre plates. A standard protocol was used [53]. Serial dilutions of the compounds were added to the test microtitre plates. Plates were incubated at 30°C for 1-2 days. Inhibition of growth was visually compared with control wells containing no drugs.

For high throughput phenotypic screenings, approximately 4700 *MATa *haploid gene deletion strains of *S. cerevisiae *in the BY4742 (*MATa ura3*Δ*0 leu2*Δ*0 his3*Δ*1 met15*Δ*0*) parental strain were maintained in an ordered array of approximately 384 individual strains in 16 plates. Gene deletion mutants were arrayed using a BioRAD colony arrayer robot or a V&P hand-held arrayer on to agar plates with sub-inhibitory concentrations of 3-AT (22 mg/ml), paromomycin (10 mg/ml), cycloheximide (45 ng/ml), streptomycin (40 mg/ml) and neomycin (5.5 mg/ml) or without drugs (control) in a method similar to that described by Parsons et al. [9] and as previously described [11]. After 1-2 days incubation at 30°C, digital images of the plates were captured and analyzed as previously described [23] with some modifications. In brief, images were converted to black (media) and white (colonies) and segmented using threshold values derived from Otsu's approach [54]. Objects empirically determined to be smaller than 0.00025 of the total white pixels in a plate were considered artifacts and eliminated. Colonies were ordered on the basis of local centers and area maps. The average value of white pixels *S_ave _*(average colony size) for each plate *Pn *was calculated from equation (1) where *N *was the total number of colonies and *S _i _*was the area of object *i *in plate *Pn*.

(1) $$S_{ave\;Pn}=1/N\sum_{i=1}^{N}S_{i\;Pn}$$

The relative size of each colony was calculated by subtracting the *S_ave Pn _*from the ordered array area explained in equation (2) for each plate.

(2) $$\Delta S_{i}=S_{i}-S_{ave\;Pn};i=1,\ldots ,384$$

The relative size of colonies calculated in this way was used to determine relative growth differences for each colony under different experimental conditions (that is, treated versus control); each experiment was repeated three times. Colonies that demonstrated 30% or more reduction in two replicates, or those with an average reduction of more than 20% (with internal variation of 20% or less) in all three experiments, were classified as "hits" or sensitive colonies.

Sensitivities of selected mutant strains identified in primary screens were confirmed by spot test analysis. Yeast cells were grown in YPD or SC liquid media to mid-log phase and diluted to a concentration of 10^-3 ^to 10^-6 ^cells/20 μl. From each dilution, 20 μl was spotted on to medium containing sub-inhibitory concentrations of the drugs or without drugs (control). The growth patterns were compared after 1-2 days at 30°C as described by Jessulat et al. [55]. Each experiment was repeated a minimum of three times.

### High throughput synthetic genetic interaction and phenotypic suppression analysis

Gene deletions in the Matα (Y7092) strain were generated by PCR-based gene targeting [56]. The 5'- and 3'-flanking regions of the target gene (55 bp for either end) and nourseothricin (NAT) resistance marker [57] were amplified. The PCR product was directly used to transform yeast cells. Transformants were selected on YPD medium containing 150 μg/ml NAT. Proper integration of the deletion cassette was confirmed by PCR.

Synthetic genetic array analyses were carried out as described previously [4]. Briefly, each query strain carrying a target gene deletion in a MATα background was crossed with a set of 384 yeast deletion mutants (MATa) known to be involved in translation. Diploids were sporulated and the resulting haploids were grown on selective plates. After several selection steps, MATa haploids carrying double gene mutations were selected. The sickness of double gene mutants was evaluated by comparing their relative quantified growth (colony size) with those for single gene mutant strains using GD software [23]. For synthetic sickness, growth differences of 30% or more were selected as positive. For alleviating interactions, growth differences of 20% or more were selected. The identified synthetic genetic interactions were confirmed by random spore analysis [58].

Gene overexpression constructs are described by Sopku et al. [59]. Suppression analysis was performed as before [18]. Briefly, overexpression plasmids were transformed into a MATα strain. The transformed strains were crossed with a set of yeast gene deletion strains for 384 translation genes as above. The sensitivities of yeast strains containing the overexpression constructs were compared to those with a control plasmid or no plasmid, against neomycin and streptomycin, using colony size measurements. A cut off value of 30% or more was used.

### Genetic assays

Alterations in translation fidelity were measured using plasmids pUKC817 and pUKC818, which carry the premature stop mutations UAA and UGA, respectively, in a β-galactosidase expression cassette. pUKC815 contains no premature stop codon and was used as a control. β-galactosidase was assayed using O-nitrophenyl-α-D-galactopyranoside (ONPG) as described previously [60] with some modification. Briefly, mutant cells were grown overnight in minimal media. Subcultures were grown to the exponential phase (OD_600 _~1.0), cells were collected by centrifugation and were resuspended in Z-buffer. The units of enzyme activity were calculated as nanomoles and represent the level of ONPG hydrolyzed per microgram of total protein [61]. All assays were conducted in triplicate. Real-time PCR analysis was performed using Rotor Gene RG-300 from Corbett Research as described [18]. Yeast total RNA was isolated using a Bio-Rad total RNA extraction kit. The total RNA was quantified by monitoring absorbance at 260 nm. cDNA was synthesized using 0.5 μg of total extracted RNA from each of the strains using reverse transcriptase (Bio-Rad) according to the manufacturer's instructions at 42°C for 45 min, and the reaction was stopped by 5 min incubation at 85°C.

The rate of total protein synthesis was evaluated *in vivo *by measuring the incorporation of [^35^S] methionine into the cellular proteins as previously described by Schwartz and Parker [62] with modifications. Briefly, yeast strains were grown to mid-log phase at 30°C in YPD. The cells were harvested, resuspended in pre-warmed minimal medium lacking methionine, and supplemented with 10 μCi/ml of [^35^S] methionine. The cells were incubated for 1 h at 30°C and harvested by centrifugation. The samples were washed with distilled water six times and 2 μl aliquots were collected on Whatman paper. The paper was air dried and exposed to storage phosphor screen for 1 h. The amount of radioactivity incorporated into total cellular proteins was measured by a Cyclon storage phosphor screen reader. Each experiment was repeated at least five times. Induced translation was measured using an inducible β-galactosidase reporter gene in p416 [41] plasmid after 4 h induction.

Polysome preparations were obtained according to the protocol of Foiani *et al. *[63]. Haploid yeast mutant and wild type strains were grown on YPD at 30°C to an OD_600 _of 0.8-1.0, and to a density of 2 × 10^7 ^cells/ml. Immediately, 200 μl of cycloheximide (50 μg/ml) was added, and each culture was quickly chilled in an ice water bath. Cells were harvested, washed and centrifuged at 4000 rpm for 4 min at 4°C using a Sorvall SLA-1500 rotor to separate the supernatant. Cell pellets were resuspended in 10 ml of ice-cold breaking buffer A (YA buffer: 10 mM Tris-HCl [pH 7.4], 100 mM NaCl, 30 mM MgCl_2_, cycloheximide 50 μg/ml, heparin 200 μg/ml) and centrifuged at 4000 rpm for 4 min at 4°C (Sorvall SS34 rotor) twice. Pellets were resuspended in 0.5 ml of YA buffer, lysed by vortexing with glass beads and stored at -80°C. Twenty OD_260 _units of each supernatant were fractionated on 8-48% sucrose gradients containing 50 mM Tris-acetate (pH 7.0), 50 mM NH_4_Cl, 12 mM MgCl_2_, and 1 mM dithiothreitol. The extract was centrifuged for 2.5 h at 39,000 rpm using a SW40-Ti rotor in a Beckman LE-80 K at 4°C. The polysome profiles were analyzed by monitoring the absorbance at 254 nm in a Beckman spectrophotometer.

## Abbreviations

yGDA: yeast non-essential Gene Deletion Array; CS: Colony Size; ST: Spot Test; TAE: Translation Associated Element; 3-AT: 3-amino-1, 2, 4-triazole.

## Authors' contributions

All authors contributed to the conceptual development of the project. MA and VE were involved in designing and conducting the experiments. AA helped in the initial development of the project. AG supervised the experiments. MA, MJ and AG were involved in data analysis and the writing of the manuscript. All authors read and approved the final manuscript.

## Supplementary Material

Additional file 1 **Gene deletion sensitivities to different bioactive compounds**. Click here for file

Additional file 2 **Descriptions of translation related genes that interact genetically with *TAE2*, *TAE3 *and *TAE4 *and produce a synthetic sick phenotype**. Click here for file

Additional file 3 **Descriptions of translation related genes that are phenotypically suppressed by overexpression of *TAE2 *and *TAE4 *against treatment with neomycin and/or streptomycin**. Click here for file
