# Supplementary material for: Chemical-genetic profile analysis of five inhibitory compounds in yeast
Source: BMC Chem Biol. 2010 Aug 6;10:6. doi: 10.1186/1472-6769-10-6 (PMC2925817; doi:10.1186/1472-6769-10-6)
Supplement: Additional file 2 — Descriptions of translation related genes that interact genetically with TAE2, TAE3 and TAE4 and produce a synthetic sick phenotype. [file 1472-6769-10-6-S2.DOC]

**Supplemental Table 2:** Descriptions of translation related genes that genetically interact with *TAE2*, *TAE3* and *TAE4* and produce a synthetic sick (SS: Super sick, VS: Very sick, MS: Moderately sick) or synthetic rescue (R) interaction.

| **Systematic Gene Name** | **Standard Gene Name** | **Interacting gene** | **Phenotype** | **Description and Cellular Function** |
| --- | --- | --- | --- | --- |
| **RNA Processing** | | | |  |
| *YAL026C* | *FUN38* | *TAE3* | MS | Aminophospholipid translocase protein homolog to human ATP8B1, involved in RNA processing |
| *YCR077C* | *PAT1*  *MRT1* | *TAE3* | MS | Small cytosolic ribosomal protein, involved in translation initiation and metabolic processes |
| *YDR378C* | *LSM6* | *TAE4* | SS | Component of heteroheptameric complexes, involved in mRNA processing |
| *YDR515W* | *SLF1*  *SRO99* | *TAE2 TAE4* | VS | Ribonucleoprotein involved in regulating mRNA translation, transport, processing |
| *YGL014W* | *PUF4* | *TAE3* | SS | PUF protein family member, involved in DNA metabolism and transcription |
| *YHR066W* | *SSF1* | *TAE2* | VS | Part of 66S ribosomal particle, participate in ribosome assembly and maintenance |
| *YKL068W* | *NUP100* | *TAE3* | R | Nuclear pore subunit, interact with Mex67p and involved in RNA processing |
| *YLR059C* | *REX2*  *YNT20* | *TAE4* | MS | RNA exonuclease, involved in rRNA maturation and RNA processing |
| *YLR107W* | *REX3* | *TAE3*  *TAE4* | R (*TAE3*) SS (*TAE4*) | RNA exonuclease activity, required for the maturation of RNA |
| *YMR125W* | *STO1*  *CBC1* | *TAE4* | VS | Component of the large subunit of mRNA cap protein complex, maintain RNA component in the nucleus, orthologous to mammalian CBP80 |
| *YNL001W* | *DOM34* | *TAE4* | R | Endoribonuclease activity, involved in mRNA catabolic process, translation |
| *YOL041C* | *NOP12* | *TAE2* | VS | This nucleolar protein reqiored for 25S rRNA processing, similar to Nop13p |
| *YOR017W* | *REX3* | *TAE4* | MS | RNA exonuclease, involved in RNA processing |
| *YPL119C* | *DBP1*  *LPH8* | *TAE4* | SS | ATP dependent RNA helicase, involved in translation initiation, mRNA processing |
|  | | | |  |
| **Mitochondrial Translation** | | | |  |
| *YBR212W* | *NGR1*  *RBP1* | *TAE2* | VS | RNA binding protein, regulates mitochondrion organization and biogenesis |
| *YCR046C* | *IMG1* | *TAE2* | MS | Structural constituent of mitochondrial ribosome, maintain mitochondrial genome |
| *YDR116C* | *MRPL1* | *TAE3 TAE4* | MS (*TAE3*)  SS (*TAE4*) | Large mitochondrial subunit involved in translation |
| *YDR494W* | *RSM28* | *TAE2* | R | Small subunit of mitochondrial ribosome involved in translation |
| *YMR225C* | *MRPL44*  *YMR44* | *TAE2* | VS | Mitochondrial large ribosomal subunit |
| *YNR022C* | *MRPL50* | *TAE3 TAE4* | VS (*TAE3*) MS (*TAE4*) | Large subunit of mitochondrial ribosomal protein |
| *YOR017W* | *PET127* | *TAE4* | MS | Mitochondrial membrane protein, involved in RNA processing |
|  | | | |  |
| **Translation Regulation** | | | |  |
| *YDL219W* | *DTD1* | *TAE2* | SS | D-tyrosyl-tRNA(Tyr) deacylase activity, involved in translation |
| *YDR223W* | *CRF1* | *TAE2* | SS | Transcriptional corepressor, regulate ribosomal protein gene transcription |
| *YFR009W* | *GCN20* | *TAE3 TAE4* | VS (*TAE3*) SS (*TAE4*) | Gcn20p regulate Gcn2p kinase, translation elongation activity |
| *YGL195W* | *GCN1* | *TAE2 TAE4* | MS (*TAE2*) VS (*TAE4*) | Cytosolic protein which regiulates translation elongation, Gcn2p kinase activity |
| *YHR077C* | *NMD2*  *IFS1* | *TAE2 TAE4* | MS (*TAE2*) SS (*TAE4*) | Involved in the nonsense mediated decay pathway, maintain telomere |
| *YMR080C* | *NAM7*  *IFS2* | *TAE4* | MS | ATP-dependent RNA helicase of the SFI superfamily, involved in mRNA metabolism, regulate translation termination |
| *YOL045W* | *PSK2* | *TAE2* | MS | Member of the PAS domain S/T protein kinase, regulate sugar flux and translation |
|  | | | |  |
| **Translation Factor** | | | |  |
| *YAL035W* | *FUN12* | *TAE4* | VS | Cytosolic small ribosomal subunit, GTPase activity, involved in translation initiation |
| *YJL138C* | *TIF2* | *TAE4* | VS | Member of eukaryotic translation initiation factor 4F complex |
| *YJR047C* | *ANB1*  *HYP1* | *TAE3* | SS | Translation initiation factor, involved in the formation of peptide bond and metabolic processes |
| *YKL081W* | *TEF4*  *EFC1* | *TAE2* | SS | Component of translation elongation factor 1 complex |
| *YKR026C* | *GCN3*  *AAS2* | *TAE4* | SS | Alpha subunit of the eukaryotic translation initiation factor eIF2B |
| *YKR059W* | *TIF1* | *TAE2* | R | Translation initiation factor eIF4A, identical to Tif2p |
| *YMR012W* | *CLU1*  *TIF31* | *TAE2* | VS | Eukaryotic translation initiation factor 3 complex |
|  | | | |  |
| **60S Ribosomal Subunit** | | | |  |
| *YBL087C* | *RPL23A* | *TAE2* | SS | Cytosolic large ribosomal subunit, similar to *E. coli* L14 and rat L23 |
| *YBR084C-A* | *RPL19A* | *TAE3* | MS | Large ribosomal (60S) subunit, identical to Rp119Bp and similar to rat L19 ribosomal protein |
| *YFR032C-A* | *RPL29* | *TAE2* | MS | Structural constituent of ribosome, has similarity to rat L29 ribosomal protein |
| *YIL133C* | *RPL16A*  *RPL13* | *TAE2 TAE4* | VS | Large ribosomal subunit, binds to 5.8 S rRNA, similar to *E. coli* L13 and rat L13a |
| *YLR325C* | *RPL38* | *TAE3* | SS | Large (60S) ribosomal subunit, identical to rat L38 ribosomal protein |
| *YLR344W* | *RPL26A* | *TAE2* | MS | Large ribosomal subunit, nearly identical to Rpl26Bp, similar to *E. coli* L24 and rat L26 |
| *YPL079W* | *RPL21B* | *TAE4* | SS | Large ribosomal subunit, similar to rat L21 ribosomal protein |
|  | | | |  |
| **40S Ribosomal Subunit** | | | |  |
| *YDR025W* | *RPS11A* | *TAE2 TAE4* | MS | Protein component of the small (40S) ribosomal subunit, identical to Rps11Bp, similar to *E. coli* S17 and rat S11 |
| *YDR450W* | *RPS18A* | *TAE2* | R | Cytosolic small ribosomal subunit, similar to *E. coli* S13 and rat S18 |
| *YJL190C* | *RPS22A*  *RPS24* | *TAE2 TAE4* | SS (*TAE2*) MS (*TAE4*) | Structural constituent of ribosome, has similarity with *E. coli* S8 and rat S15a ribosomal proteins |
| *YKL156W* | *RPS27A* | *TAE2* | VS | Protein component of the small (40S) ribosomal subunit; nearly identical to Rps27Bp and has similarity to rat S27 ribosomal protein |
| *YLR441C* | *RPS1A*  *RP10A* | *TAE4* | SS | Ribosomal protein 10 (rp10) of the small (40S) subunit; nearly identical to Rps1Bp and has similarity to rat S3a ribosomal protein |
| *YML063W* | *RPS1B*  *RP10B* | *TAE4* | VS | Ribosomal protein 10 (rp10) of the small (40S) subunit; nearly identical to Rps1Ap and has similarity to rat S3a ribosomal protein |
| *YOR096W* | *RPS30*  *RPS7A* | *TAE3* | MS | Ribosomal protein component of the small (40S) subunit, similar to Rps7Bp and rat S7 |
| *YOR182C* | *RPS30B* | *TAE4* | VS | Protein component of the small (40S) ribosomal subunit; nearly identical to Rps30Ap and has similarity to rat S30 ribosomal protein |
| *YPR132W* | *RPS23B* | *TAE2* | VS | Cytosolic small ribosomal subunit, identical to Rps23Ap, similar to *E. coli* S12 and rat S23 |
|  | | | |  |
| **Amino Acid Metabolism** | | | |  |
| *YER091C* | *MET6* | *TAE2* | VS | Methyl transferase activity, involved in amino acid metaboli process |
| *YIL074C* | *SER33* | *TAE2* | SS | Phophoglycerate dehydrogenase activity, involved in the biosynthesis of serine family amino acids |
| *YJR130C* | *STR2* | *TAE2* | VS | Cystathionine gamma-synthase activity, involved in amino acid metabolism |
| *YLR180W* | *SAM1*  *ETH10* | *TAE4* | MS | S-adenosimemethionine synthetase, involved in methionine metabolic processes |
| *YNL277W* | *MET2* | *TAE2* | SS | L-homoserine-O-acetyltransferase, catalyzes the conversion of homoserine to O-acetyl homoserine which is the first step of the methionine biosynthetic pathway |
| *YOR184W* | *SER1*  *ADE9* | *TAE2* | VS | 3-phosphoserine aminotransferase, catalyzes the formation of phosphoserine from 3-phosphohydroxypyruvate, required for serine and glycine biosynthesis |
|  | | | |  |
| **Others** | | | |  |
| *YFL031W* | *SNF4*  *CAT3* | *TAE3* | SS | AMP-activated Snf1p protein kinase complex, involved in sporulation, and peroxisome biogenesis |
| *YGL248W* | *PDE1* | *TAE2* | SS | 3',5'-cyclic-AMP phosphodiesterase activity, induce transcription |
| *YGR166W* | *KRE11*  *TRS65* | *TAE4* | MS | Protein involved in the biosynthesis of cell wall component |
| *YLR085C* | *APR6* | *TAE4* | MS | Nucleosome binding protein, involved in DNA metabolic processes |
| *YNL298W* | *CLA4*  *ERC10* | *TAE2* | SS | Cdc42p activated signal transducing kinase of the p21-activated kinase family, involved in septin ring assembly and cytokinesis |
| *YNL299W* | *TRF5* | *TAE2* | MS | Poly (A) polymerase involved in nuclear RNA quality control based on: homology with Trf4p |
