# Supplementary material for: Chemical-genetic profile analysis of five inhibitory compounds in yeast
Source: BMC Chem Biol. 2010 Aug 6;10:6. doi: 10.1186/1472-6769-10-6 (PMC2925817; doi:10.1186/1472-6769-10-6)
Supplement: Additional file 3 — Descriptions of translation related genes that are phenotypically suppressed by overexpression of TAE2 and TAE4 against treatment with neomycin and/or streptomycin. [file 1472-6769-10-6-S3.DOC]

**Supplemental Table 3**: Descriptions of translation related genes that are phenotypically suppressed by overexpression of *TAE2* and *TAE4* against treatment with neomycin and/or streptomycin.

| **Gene deletion strains** | | **Treatment** | | **Description and Cellular Function** |
| --- | --- | --- | --- | --- |
| **Systematic**  **gene name** | **Standard gene name** | **Neomycin** | **Streptomycin** |
|  | | | |  |
| ***TAE2* OVEREXPRESSION** | | | |  |
|  | | | | |
| **Translation Factor** | | | | |
| *YKL204W* | *EAP1* |  | Complete | eIF4E-associated protein, maintain genetic stability |
| *YKR059W* | *TIF1* | Complete |  | Eukaryotic translation initiation factor eIF4A |
| *YMR012W* | *CLU1*  *TIF31* |  | Complete | Translation initiation factor, eIF3 component of unknown function |
| *YOL023W* | *IFM1* | Partial |  | Mitochondrial translation initiation factor 2 |
|  | | | | |
| **Mitochondrial Translation** | | | | |
| *YDR462W* | *MRPL28* |  | Partial | Mitochondrial ribosomal protein of the large subunit|Mrpl28p |
| *YKL167C* | *MRP49* | Partial |  | Structural constituent of mitochondrial ribosomal proten |
| *YKR006C* | *MRPL13* | Partial |  | mitochondrial large ribosomal subunit |
| *YMR012W* | *CLU1*  *TIF31* |  | Complete | Translation initiation factor, eIF3 component of unknown function |
| *YOL023W* | *IFM1* | Partial |  | Mitochondrial translation initiation factor 2 |
|  | | | | |
| **Large (60S) ribosomal subunit** | | | | |
| *YBL027W* | *RPL19B* | Complete | Complete | Large (60S) ribosomal subunit, identical to Rpl19Ap and similar to rat L19 ribosomal protein |
| *YDL184C* | *RPL41A*  *RPL47A* | Complete |  | Ribosomal protein L47 of the large (60S) ribosomal subunit, identical to Rpl41Bp and has similarity to rat L41 ribosomal protein |
| *YER056C-A* | *RPL34A* | Partial |  | Protein component of the large (60S) ribosomal subunit, nearly identical to Rpl34Bp and has similarity to rat L34 ribosomal protein |
| *YIL052C* | *RPL34B* |  | Complete | Cytosolic large (60S) ribosomal subunit, nearly identical to Rpl34Ap and has similarity to rat L34 ribosomal protein |
| *YMR242C* | *RPL20A*  *RPL18A2* | Complete | Partial | Protein component of the large (60S) ribosomal subunit, nearly identical to Rpl20Bp and has similarity to rat L18a ribosomal protein |
| *YOR312C* | *RPL20B*  *RPL18A1* | Complete |  | the large (60S) ribosomal subunit, nearly identical to Rpl20Ap and has similarity to rat L18a ribosomal protein |
|  | | | | |
| **Small (40S) ribosomal subunit** | | | | |
| *YDL083C* | *RPS16B* | Complete |  | The small (40S) ribosomal subunit; identical to Rps16Ap and has similarity to E. coli S9 and rat S16 ribosomal protein |
| *YPL081W* | *RPS9A* |  | Complete | Cytosolic small (40S) ribosomal subunit; nearly identical to Rps9Bp and has similarity to E. coli S4 and rat S9 ribosomal proteins |
|  | | | | |
| **Others** | | | | |
| *YER081W* | *SER3* |  | Complete | 3-phosphoglycerate dehydrogenase, involved in serine and glycine biosynthesis |
| *YFL001W* | *DUG1*  *PUS3* | Partial | Partial | tRNA-pseudouridine synthase activity |
| *YGR155W* | *CYS4*  *NHS5* |  | Complete | Cystathionine beta-synthase, catalyzes the synthesis of cystathionine from serine and homocysteine |
| *YGR285C* | *ZUO1* | Complete |  | Cytosolic ribosome-associated chaperone |
| *YIL074C* | *SER33* |  | Complete | 3-phosphoglycerate dehydrogenase, catalyzes the first step in serine and glycine biosynthesis |
|  | | | |  |
| ***TAE4* OVEREXPRESSION** | | | |  |
|  | | | | |
| **RNA processing** | | | | |
| *YDL213C* | *NOP6* |  | Partial | Putative RNA-binding protein implicated in ribosome biogenesis |
| *YER032W* | *FIR1*  *PIP1* |  | Complete | Protein involved in 3' mRNA processing |
| *YGR159C* | *NSR1* | Complete |  | Nuclear protein, required for pre-rRNA processing and ribosome biogenesis |
| *YJL124C* | *LSM1*  *SPB8* | Complete |  | small nucleolar ribonucleoprotein forms heteroheptameric complex |
| *YPL178W* | *CBC2*  *CBP20* |  | Partial | Small subunit of the heterodimeric cap binding complex |
|  | | | | |
| **Small (40S) ribosomal subunit** | | | | |
| *YDL083C* | *RPS16B* |  | Partial | Small (40S) ribosomal subunit; identical to Rps16Ap and has similarity to E. coli S9 and rat S16 ribosomal proteins |
| *YGR118W* | *RPS23A* |  | Complete | Ribosomal protein 28 (rp28) of the small (40S) ribosomal subunit, required for translational accuracy |
| *YGR214W* | *RPSOA*  *NAB1* | Complete |  | Cytosolic protein component of the small (40S) ribosomal subunit, nearly identical to Rps0Bp; required for maturation of 18S rRNA |
| *YKL156W* | *RPS27A* | Partial |  | Protein component of the small (40S) ribosomal subunit; nearly identical to Rps27Bp and has similarity to rat S27 ribosomal protein |
| *YKR057W* | *RPS21A*  *RPS25* | Complete |  | Small (40S) ribosomal subunit; nearly identical to Rps21Bp and has similarity to rat S21 ribosomal protein |
| *YLR367W* | *RPS22B* | Complete |  | Protein component of the small (40S) ribosomal subunit; nearly identical to Rps22Ap and has similarity to E. coli S8 and rat S15a ribosomal proteins |
| *YML026C* | *RPS18B* | Partial |  | Small ribosomal subunit, nearly identical to Rps27Bp and similar to rat S27 ribosomal protein |
| *YMR143W* | *RPS16A* | Partial | Complete | Protein component of the small (40S) ribosomal subunit; identical to Rps16Bp and has similarity to E. coli S9 and rat S16 ribosomal proteins |
| *YPL081W* | *RPS9A* |  | Partial | Small (40S) ribosomal subunit; nearly identical to Rps9Bp and has similarity to E. coli S4 and rat S9 ribosomal proteins |
|  | | | | |
| **Others** | | | | |
| *YDL075W* | *RPL31A*  *RPL34* | Complete |  | Large (60S) ribosomal subunit, nearly identical to Rpl31Bp and has similarity to rat L31 ribosomal protein |
| *YIL052C* | *RPL34B* |  | Partial | Protein component of the large (60S) ribosomal subunit, nearly identical to Rpl34Ap and has similarity to rat L34 ribosomal protein |
| *YOR302W* | None | Complete |  | Arginine attenuator peptide, regulates translation of the CPA1 mRNA |
| *YPR189W* | *SKI3* |  | Complete | Protein involved in exosome mediated 3' to 5' mRNA degradation and translation inhibition of non-poly(A) mRNAs |
